# Umbilical cord mesenchymal stem cells improve bone regeneration in diabetes mellitus animal model with apical periodontitis

**DOI:** 10.1016/j.jobcr.2024.11.009

**Published:** 2024-11-29

**Authors:** Eric Priyo Prasetyo, Pravinna Saravanan, Deaniddo Kharisna, Christina Immee Wijanarko, Mefina Kuntjoro, Nike Hendrijantini, Evelyn Tjendronegoro

**Affiliations:** a Department of Conservative Dentistry, Faculty of Dental Medicine, Universitas Airlangga, Surabaya, Indonesia; b Department of Prosthodontics, Faculty of Dental Medicine, Universitas Airlangga, Surabaya, Indonesia; c Healthcare and Research, Irvine Medical Center, University of California, Irvine, United States

**Keywords:** Apical periodontitis, Bone regeneration, Diabetes mellitus, Endodontic treatment, Mesenchymal stem cells

## Abstract

**Background:**

Previous studies revealed diabetes mellitus subjects tend to have persistent apical periodontitis. Regenerative stem cells therapy through endodontic procedure is hoped to be a solution. This study assessed bone regeneration in diabetic rats with apical periodontitis through histopathological analysis of osteoblasts and immunohistochemical analysis of runt-related transcription factor 2 (Runx2) and Osterix.

**Methods:**

Diabetes mellitus and apical periodontitis was induced on 20 rats. Apical periodontitis was induced on mandibular right first molars under anesthesia. The teeth were left open for 7 days following access cavity and pulp extirpation, then the rats’ teeth were endodontically treated and randomly allocated into 4 groups (5 rats per group). The first and second groups was ended at 30 days (C30) and 60 days (C60) and labelled as control. The third and fourth groups was given umbilical cord mesenchymal stem cells and ended at 30 days (T30) and 60 days (T60). The osteoblasts, Runx2 and Osterix were analyzed. ANOVA and Tukey HSD tests were used for analysis. Differences with p values < 0.05 were considered significant.

**Results:**

The number of osteoblasts in the apical area in control groups (C30 and C60) and treatment groups (T30 and T60) showed a significant increase (p < 0.05). The expressions of Runx2 and Osterix in osteoblasts showed a significant increase among the control (C30 and C60) and treatment groups (T30 and T60) (p < 0.05).

**Conclusion:**

Umbilical cord mesenchymal stem cells improve bone regeneration in diabetic animal model with apical periodontitis, in terms of osteoblasts, Runx2 and Osterix.

## Graphical abstract

**Figure:**
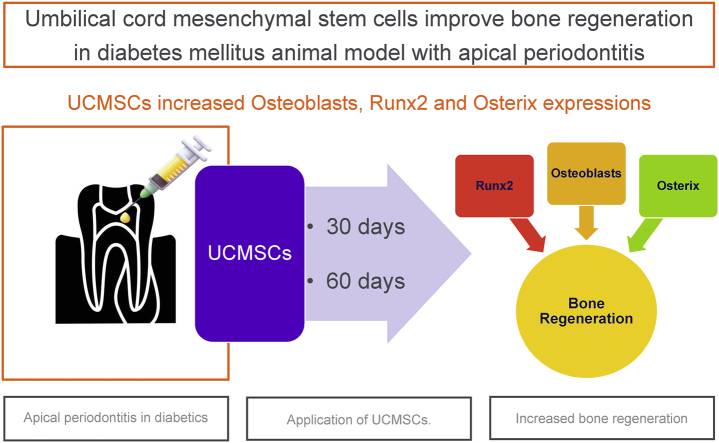

## 1 Introduction

Diabetes mellitus (DM) is a global problem involving all age groups. It is a degenerative disease featured by hyperglycemia as a result from lacks of insulin secretion or reactiveness and the population suffering this metabolic disease is predicted to rise beyond 600 million worldwide population by 2040.^1^ Long term unmedicated hyperglycemia would cause macro and micro vascular changes in the oral tissues.^2^ Unregulated hyperglycemia would increase problems in the oral region for instance caries, periodontal problems, tooth mobility, pulp necrosis, apical periodontitis and tooth loss.^3^^,^^4^

Apical periodontitis (AP) is an inflammation within the apical site, prompting inflammation, alveolar bone resorption and destruction.^5^ This inflammation influence tissues in the apical site and involve host defense mechanism to produce pro-inflammatory cytokines.^6^ These cytokines in hyperglycemia condition are accountable for increased level of chronic inflammation, prolonged apical tissue healing and accelerate bone damage.^7^ In the mechanism of AP and bone destruction in subjects with DM, macrophages and neutrophils are important cells in directing inflammation and bone ruin, altogether with the production of inflammatory mediators affecting osteoclasts activation and bone resorption.^8^

Stem cells has been explored for their advantages for tissue regeneration. This supports the use of stem cells in regenerative endodontic therapy (RET). Human umbilical cord mesenchymal stem cells as one of many mesenchymal stem cells sources could be obtained and cultured easily with high proliferation. In-vitro experiments have been done to study these cells.^9^, ^10^, ^11^ Compared to other sources, mesenchymal stem cells from umbilical cord are more primitive, immune regulatory, and rich in stemness. These cells have a potential use in regenerative therapy.^12^, ^13^, ^14^ Application of umbilical cord mesenchymal stem cells (UCMSCs) in endodontics are emerging and needs to be explored.

Clinical findings and several previous studies showed that diabetes mellitus will have a negative impact on periapical bone regeneration, even after adequate endodontic treatment.^15^^,^^16^ Osteogenic differentiation in bone regeneration process is regulated by transcription factors, such as runt related transcription factor 2 (Runx2) and Osterix.^17^ In this study, we studied whether UCMSCs induction in diabetic rats with apical periodontitis will positively affect bone regeneration by evaluating the changes in the number of osteoblasts and the expressions of Runx2 and Osterix after 30 and 60 days. Our proposed hypothesis is UCMSCs application in diabetic rats with apical periodontitis will increase the number of osteoblasts, Runx2 and Osterix expressions after 30 and 60 days.

## 2 Materials and methods

### 2.1 Animals

This study was a randomized post-test only true experimental design. All experiment involving animals were conducted according to relevant guidelines and regulations, including National Research Council's guide for the care and use of laboratory animals. The experiment was reviewed and ethically cleared by the Health Ethic Commissions, Universitas Airlangga Faculty of Dental Medicine (Document no. 1170/2023). Twenty adult male rats (Wistar strain*, Rattus norvegicus*), healthy, aged 10–13 weeks, weighing 250–300 g were used in this study. Sample calculation was determined based on previous study. The rats were randomly divided into 4 groups with 5 rats each, being 4 investigational groups (C30: control of 30 days, C60: control of 60 days, T30: treatment of 30 days, and T60: treatment of 60 days). The rats were kept in diurnal lighting condition (12 h dark/light cycle) with 45–55 % relative humidity and 25 °C temperature. Rats were under the care of veterinarians, given free admittance to water and food ad libitum.

### 2.2 Preparation of diabetes mellitus model using streptozotocin

Rat model of diabetes mellitus was done following previous method.^18^ In brief, blood glucometer (AccuCheck, Germany) was operated to check the rats’ blood glucose level previously, throughout, and five days afterwards the end of streptozotocin (Bioworld, USA) injections. Streptozotocin (STZ) was dissolved in buffer citrate 0.05M, pH 4.5. Six hours beforehand daily STZ intraperitoneal injection (20 mg/kg body weight), the rats were fasted. Every after STZ injections, glucose solution 5 % (Otsuka, Indonesia) were provided for 24 h. Confirmation of positive DM was done one week afterwards STZ injection by checking the blood glucose of over 300 mg/dL.

### 2.3 Preparation of apical periodontitis by root canal exposure to oral cavity

After successful DM induction was confirmed on the rats, initiation for apical periodontitis was conducted according to previous method with modification.^19^ In short, adult male rats weighing 250–300 g were given intraperitoneal anesthesia injection comprising 40 mg/kg ketamine hydrochloride (Kepro B.V., The Netherlands) and 5 mg/kg xylazine (Xyla Interchemie, The Netherlands). Mandibular right first molars were prepared with diamond round bur no 1 (SS White, USA) to gain access cavity, then pulpectomy procedure were conducted using barbed brooch (Dentsply, USA). The teeth were left open for communication to oral microorganisms.

### 2.4 Preparation of umbilical cord mesenchymal stem cells

All experiment involving humans were conducted according to relevant guidelines and regulations, including ethical principles for medical research involving human subjects as stated in the Declaration of Helsinki by the World Medical Association and World Health Organization guiding principles on human cell, tissue, and organ transplantation. The UCMSCs were taken from frozen stock provided by the Universitas Airlangga Stem Cells Research and Development Center, Surabaya, Indonesia, from medical waste of donor with written informed consent. In short, after UCMSCs confirmation by flow cytometry (positive CD90, CD105, CD73, and negative CD34, CD45), stem cells of passage 5 were used. Gelatin was used as scaffold material to carry 500,000 cells for injection into the root canals and pulpal space. Conical tubes carrying 20 μL UCMSCs in gelatin scaffold were prepared for injection.

### 2.5 Application of umbilical cord mesenchymal stem cells into the root canals

Seven days after apical periodontitis induction, preparation of the root canal was performed using K-file number 8, 10 and 15 (Dentsply, USA). Digital apex locator was utilized (Dentsply, USA) to support preparation. Irrigation of sodium hypochlorite 1.5 % (Hyposol, India) was carried out following root canal preparation. Final irrigation was done using sterile saline (Otsuka, Indonesia). Canal debridement and irrigation was performed after canal preparation with Navi-tips (Ultradent, USA). The canals were dried using sterilized paper points (Dentsply, USA) number 15. The arranged 20 μL UCMSCs in gelatin scaffold was delivered into the root canal with a microliter syringe (Hamilton, USA). The coronal part was covered with bio-c repair (Angelus, Brazil) and covered with glass ionomer restoration (GC, Japan). The rats were terminated on 30 and 60 days afterwards injection of UCMSCs through anesthetic overdose (euthanasia) injection with xylazine 30 mg/kg and ketamine hydrochloride 300 mg/kg intraperitoneally. The mandible specimens were collected and soaked in paraformaldehyde 10 %.

### 2.6 Histopathological and immunohistochemical analysis

The specimens were decalcified using ethylene diamine tetra acetic acid (EDTA) aqueous solution 5 % (Merck, USA), embedded in paraffin slabs (Pro Histo, Indonesia) then cut by a microtome (Leica RM 2125 RTS, Germany) using microtome blades (Diacut Ultra Plus Microtome Blades, Italy) at 5 μm thickness according to standard protocol. Hematoxylin Eosin (HE) staining with Mayer's Hematoxylin (Merck, USA) and Eosin (Merck, USA) was used for histopathological examination to assess osteoblasts. Segments of involved teeth with apical periodontitis were attended.

Immunohistochemical stain was conducted by anti Runx2 (SC101145, Santa Cruz Biotechnology, USA) and anti Osterix (AB209484, Abcam, USA) monoclonal antibody to assess bone regeneration. For the number of osteoblasts and osteoclasts, a standardized guide was lain on captured images. The cells were counted in 100 μm squares in five fields around periapical region.

Microscopically in the apical area of apical periodontitis, osteoblasts were counted from HE stained specimens, Runx2 and Osterix were counted from IHC stained specimens. Immunohistochemical and histopathological analysis were performed using a light microscope Nikon Eclipse Ci-E compound microscope (Nikon Corp, Japan) at 400× magnification concentrating in the apical area of apical periodontitis.

### 2.7 Data analysis

The data were gathered and computed as mean ± standard deviation. Statistical analysis was processed using SPSS software (SPSS, version 27, SPSS Inc., IBM, NY, USA). P value of less than 0.05 was considered statistically significant. Data were assessed using analysis of variance (ANOVA) test and continued by post-hoc comparisons between groups.

## 3 Results

### 3.1 Blood glucose level

The blood glucose of all rats before STZ treatment were measured normally (70–130 mg/dL). Throughout the STZ injection, blood glucose was increased variably (140–320 mg/dL), and afterwards the final of 5 days induction, the blood glucose was steady around 300–490 mg/dL. The blood glucose stays steady afterwards. All of the rats survived the multiple low doses of STZ.

### 3.2 Osteoblasts

**Fig. 1:**
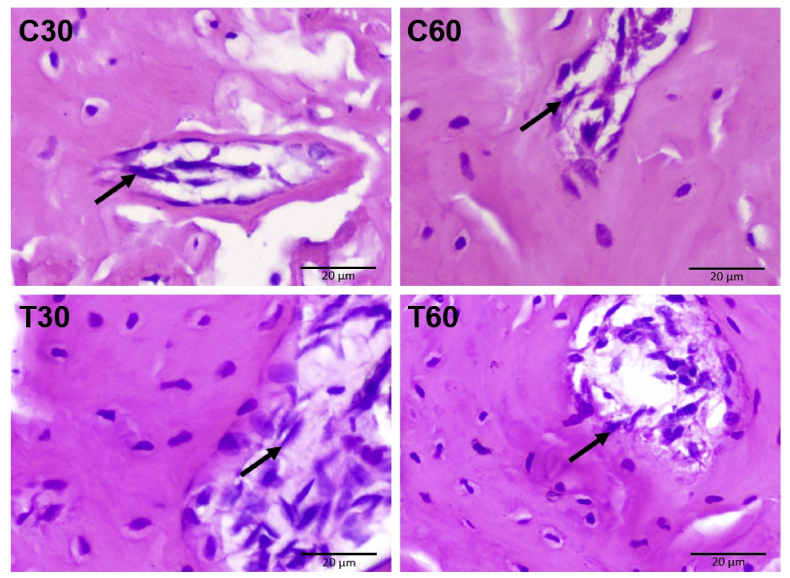
Microscopic examination on the number of osteoblasts found in the apical area are indicated by black arrow (C30: control group of 30 days, C60: control group of 60 days, T30: UCMSCs treatment group of 30 days, T60: UCMSCs treatment group of 60 days). Scale bar: 20 μm.

Examination on the specimens on the number of osteoblasts found in the apical area was performed on control and treatment groups. Hematoxylin Eosin staining is shown in Fig. 1. The data were normal and the variances were homogeny (p > 0.05). The result of osteoblast count in the control group of 30 days (C30) was 6.56 ± 2.47; in the control group of 60 days (C60) was 7.00 ± 1.82; in UCMSCs treatment group of 30 days (T30) was 13.08 ± 1.58; in UCMSCs treatment group of 60 days (T60) was 13.68 ± 2.05. The treatment groups with UCMSCs significantly lead the number of osteoblasts both in T30 and T60, while the control groups of C30 and C60 remain low (p < 0.05). The number of osteoblasts between the control groups of 30 and 60 days (C30 and C60) were not significantly different (p = 0.985). The number of osteoblasts between UCMSCs treatment groups of 30 and 60 days (T30 and T60) were not significantly different (p = 0.964). Significant difference in osteoblast count were found between C30 and T30 (p = 0.001); C30 and T60 (p = 0.000); C60 and T30 (p = 0.001); C60 and T60 (p = 0.000). The mean and standard deviation of osteoblasts count is available in Table 1. The significance of osteoblasts count compared among groups is available in Table 2.

**Table 1:** Mean and standard deviation of osteoblasts count, Runx2, and Osterix expressions among groups.

Groups	Osteoblasts count	Runx2 expression	Osterix expression
Control 30 days (C30)	6.56 + 2.47	4.24 ± 0.94	3.48 ± 1.20
Control 60 days (C60)	7.00 + 1.82	3.04 ± 0.82	4.12 ± 1.06
UCMSCs 30 days (T30)	13.08 + 1.58	10.88 ± 1.58	9.08 ± 1.68
UCMSCs 60 days (T60)	13.68 + 2.05	8.20 ± 1.55	9.04 ± 1.47

**Table 2:** Significance of osteoblasts count, Runx2, and Osterix expressions among groups.

Comparisons	Osteoblasts count	Runx2 expression	Osterix expression
Control 30 to Control 60 days	p = 0.985	p = 0.463	p = 0.881
Control 30 to UCMSCs 30 days	p = 0.001^a^	p = 0.000^a^	p = 0.000^a^
Control 30 to UCMSCs 60 days	p = 0.000^a^	p = 0.001^a^	p = 0.000^a^
Control 60 to UCMSCs 30 days	p = 0.001^a^	p = 0.000^a^	p = 0.000^a^
Control 60 to UCMSCs 60 days	p = 0.000^a^	p = 0.000^a^	p = 0.000^a^
UCMSCs 30 to UCMSCs 60 days	p = 0.964	p = 0.020^a^	p = 1.000

a Significant difference.

### 3.3 Osteogenic expression in the apical area

**Fig. 2:**
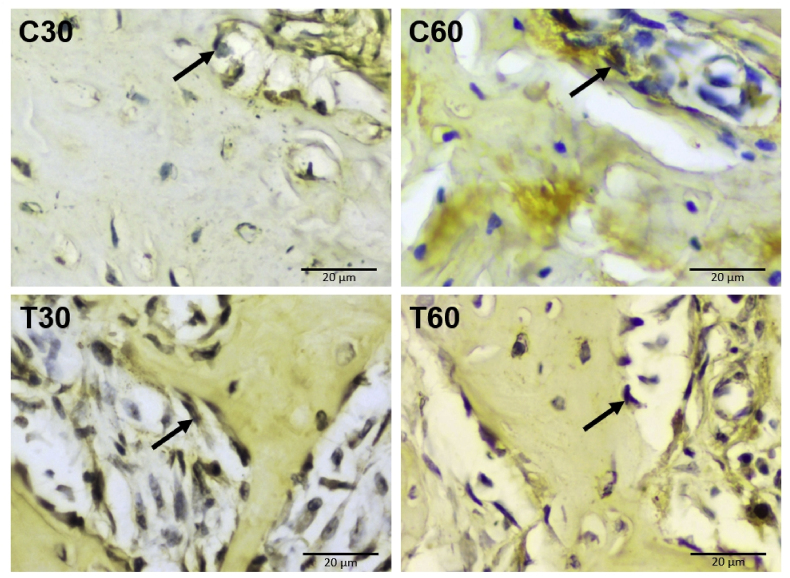
Runx2 expression in osteoblasts. Surface immune reactivity of osteoblasts in the apical area are indicated by black arrow (C30: control group of 30 days, C60: control group of 60 days, T30: UCMSCs treatment group of 30 days, T60: UCMSCs treatment group of 60 days). Scale bar: 20 μm.

**Fig. 3:**
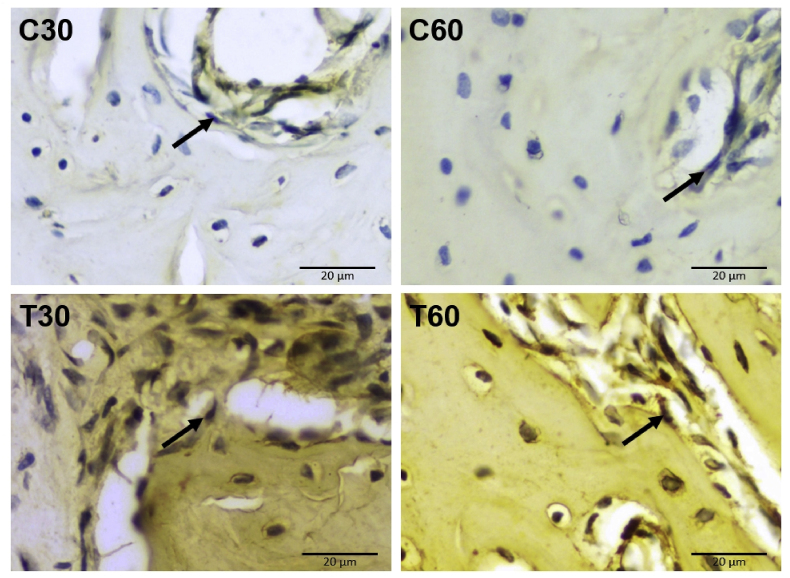
Osterix expression in osteoblasts. Surface immune reactivity of osteoblasts in the apical area are indicated by black arrow (C30: control group of 30 days, C60: control group of 60 days, T30: UCMSCs treatment group of 30 days, T60: UCMSCs treatment group of 60 days). Scale bar: 20 μm.

Immunohistochemical staining examination of osteogenic expressions were done to the specimens. Osteoblast expression of runt-related transcription factor 2 (Runx2) observed at 30 and 60 days is shown in Fig. 2. Osteoblast expression of Osterix observed at 30 and 60 days is shown in Fig. 3.

Runx2 expression in the control group of 30 days (C30) was 4.24 ± 0.94; in the control group of 60 days (C60) was 3.04 ± 0.82; in UCMSCs treatment group of 30 days (T30) was 10.88 ± 1.58; in UCMSCs treatment group of 60 days (T60) was 8.20 ± 1.55. The treatment groups with UCMSCs significantly lead Runx2 expression both in T30 and T60, while the control groups of C30 and C60 remain low (p < 0.05). Runx2 expression between the control groups of 30 and 60 days (C30 and C60) were not significantly different (p = 0.463). Runx2 expression between UCMSCs treatment groups of 30 and 60 days (T30 and T60) were significantly different (p = 0.020). Significant difference in Runx2 expression were found between C30 and T30 (p = 0.000); C30 and T60 (p = 0.001); C60 and T30 (p = 0.000); C60 and T60 (p = 0.000). The mean and standard deviation of Runx2 expression among groups is available in Table 1. The significance of Runx2 expression compared among groups is available in Table 2.

Osterix expression in the control group of 30 days (C30) was 3.48 ± 1.20; in the control group of 60 days (C60) was 4.12 ± 1.06; in UCMSCs treatment group of 30 days (T30) was 9.08 ± 1.68; in UCMSCs treatment group of 60 days (T60) was 9.04 ± 1.47. The treatment groups with UCMSCs significantly lead Osterix expression both in T30 and T60, while the control groups of C30 and C60 remain low (p < 0.05). Osterix expression between the control groups of 30 and 60 days (C30 and C60) were not significantly different (p = 0.881). Osterix expression between UCMSCs treatment groups of 30 and 60 days (T30 and T60) were not significantly different (p = 1.000). Significant difference in Osterix expression were found between C30 and T30 (p = 0.000); C30 and T60 (p = 0.000); C60 and T30 (p = 0.000); C60 and T60 (p = 0.000). The mean and standard deviation of Osterix expression among groups is available in Table 1. The significance of Osterix expression compared among groups is available in Table 2.

## 4 Discussion

Animal modeling to assess DM and apical periodontitis using rats is well established. Past experiments have shown the progression of periapical tissue is comparable to those presented in humans. Streptozotocin use for reproducible diabetes mellitus induction in rats would create similar metabolism as diabetes mellitus in humans.^20^ Rats have similar histological structures, therefore, the mechanism of apical periodontitis in rats is comparable to that observed in humans. Our study presented a model on diabetes mellitus rats using open pulp and root canal exposure to allow infection from oral microorganisms which trigger apical periodontitis.

Regeneration of periodontal tissue in the apical area is a coordinated mechanism concerning inter-related events, involving cytokine and growth factor release, cells migration and proliferation.^19^ Diabetes mellitus influence the quality, quantity and functions of endogenous mesenchymal stem cells instigated by the accumulated advanced glycation end products.^3^ therefore, these cells are seldom used as autologous therapy.^21^ Diabetic subjects suffers from inferior bone vascularization, niche, and endogenous stem cells in affected areas lead to incomplete and delayed bone regeneration.^22^

Osterix and runt related transcription factor 2 (Runx2) are both prominent transcription factors in bone regeneration. Osterix is a specific transcription factor expressed by osteoblasts which is essential for osteoblastic differentiation and maturation.^23^ Osterix deficiency would cause the absence of osteoblasts and bone formation. Osterix induce pre-osteoblasts differentiation into mature and functional osteoblasts.^24^ Osterix expression in diabetes mellitus subjects is low.^25^ In normal subjects, Osterix expression is high, particularly in the periapical area of alveolar bone.^26^

The function of Runx2 in bone regeneration could be direct or through Runx2 related signaling pathways.^23^ Patients with DM exhibited a decreased amount of Runx2 expression and this significantly decrease bone density and trabecular volume.^27^ Runx2 is essential to control osteocalcin expression and inhibit mesenchymal differentiation into adipogenicity pathways caused by diabetic conditions.^28^ Runx2 have a direct correlation with persistent periapical inflammation and bone regeneration after endodontic treatment.^29^ Runx2 also participate in bone mineralization through interaction with other factors, such as alkaline phosphatase, Osterix, osteocalcin, by regulating osteoblast differentiation, matrix construction, and mineralization throughout bone development.^30^

Bone regeneration in the apical area is an orchestrated process which involve osteoblasts, Runx2 and Osterix transcription factors activated by specific osteogenic signals to produce and construct matured and functional bone. In our study, UCMSCs application in the root canal successfully increase Runx2 and Osterix expressions. Application of UCMSCs also increase the number of osteoblasts both in 30 and 60 days. Without UCMSCs application, Runx2 and Osterix expressions remain low. Runx2 expression in 60 days observation is significantly higher than 30 days observation. This condition is related to diabetes mellitus condition where constant hyperglycemia has an important part in bone healing.

DM in the host system enable apical periodontitis to persist and lead to bone destruction. In both diabetes mellitus type 1 and type 2, hyperglycemia alter immune system, increase the prevalence of apical periodontitis, increase asymptomatic infection and osteolytic lesions.^8^ Molecularly, hyperglycemia is a stimulus of bone resorption, inhibit osteoblastic differentiation, and decrease bone healing.^31^ Application of UCMSCs in the root canal would affect the local apical milieu into regeneration. The UCMSCs may advance microenvironment in the apical area because these cells can proliferate, differentiate, and immunomodulate.

Generally, the main therapies of diabetes mellitus are glucose controlling drugs and insulin injection. Both therapies are effective in maintaining blood glucose level in control but their effects on periodontal tissues require deeper study.^32^ Metformin was reported to have a positive effect on apical lesions in animal.^33^ However, this drug has gastrointestinal side effects.^34^ Previous study reported that Insulin injection does not benefit bone regeneration, metabolism, vascularization, and neuropathy.^35^ Therefore, subjects with diabetes mellitus tends to have persistent periapical problems even though endodontic treatment was performed.^15^^,^^16^

Human umbilical cord mesenchymal stem cells have some advantages, such as their ability to proliferate and differentiate into other progenitor cells, more primitive than other mesenchymal stem cells sources.^36^ Previous research with diabetic and osteoporotic animal model showed an improved bone condition.^18^^,^^37^ Our study applies exogenous mesenchymal stem cells into the root canal space with purpose to facilitate stem cells application into the apical area, and hopefully this procedure may be beneficial for cell-based regenerative endodontic therapy in subjects with diabetes mellitus. Diabetes mellitus where hyperglycemia and high amount of advanced glycation endo products remain in long period negatively affect host stem cells proliferation and differentiation.^38^ Therefore, exogenous stem cells application into the root canal space was done as a solution to cover diabetic host stem cells amount, quality, and poor differentiation.^39^

In this study, the presence of higher osteoblasts, more bone formation and maturation in the apical region indicate UCMSCs application would improve osteoblasts differentiation and increase bone regeneration. The constant number of advanced glycation end-products were associated to DM where endless hyperglycemia plays a vital part in tissue regeneration. This study accepts our hypothesis that UCMSCs application in diabetic rats with apical periodontitis will increase the number of osteoblasts, Runx2 and Osterix expressions after 30 and 60 days. However, there are certain limitations. This animal study only focuses in osteoblasts, expressions of Runx2 and Osterix, and the time ranges are limited to 30 and 60 days. Further experiments should be done to explore UCMSCs enrichments, or using other sources of mesenchymal stem cells with different observation time and other marker expressions.

## 5 Conclusion

In conclusion, our study verified that UCMSCs application into the root canal successfully increased bone regeneration in apical periodontitis under diabetes mellitus condition. Future material addition and procedures for clinical application are needed for optimal regeneration in endodontic therapy.

## Consent statement

Informed consent in both English and Bahasa Indonesia was received from donor in the study. Information sheet explaining about the aim and benefit of the study was also given to the donor. After receiving the informed consent from donor, the sample was collected.

## Data availability statement

The data that support the findings of this study are contained within the article.

## Ethical clearance

This study was ethically cleared by the Health Ethic Commissions, Universitas Airlangga Faculty of Dental Medicine (Document no. 1170/2023).

## Sources of funding

This study did not receive any specific grant from funding agencies in the public, commercial, or not-for-profit sectors.

## Declaration of competing interest

The authors declare that they have no known competing financial interests or personal relationships that could have appeared to influence the work reported in this paper.
